# Endoscopic thyroidectomy via chest-collarbone approach versus conventional open thyroidectomy: a retrospective comparative study

**DOI:** 10.1016/j.bjorl.2024.101429

**Published:** 2024-04-03

**Authors:** Yuming Lou, Lutong Liu, Miaomiao Jin, Bifei Fu, Chaoyang Xu, Xiaofeng Lu

**Affiliations:** Zhejiang University School of Medicine, Affiliated Jinhua Hospital, Department of Breast and Thyroid Surgery, Jinhua, P.R. China

**Keywords:** Thyroid cancer, Endoscopic thyroidectomy, Chest-collarbone approach

## Abstract

**Objective(s):**

Endoscopic thyroidectomy, such as axillary, areola and transoral approaches, offer the advantage of a good cosmetic outcome, but it requires a wider dissection field compared to open thyroidectomy. Recently, chest-collarbone approach thyroidectomy has been widely developed in China because of its shorter anatomical route compared to other endoscopic approaches. This study retrospectively evaluated endoscopic thyroidectomy via chest-collarbone approach in patients with thyroid nodules to determine its feasibility.

**Methods:**

A total of 46 patients with thyroid disease who underwent endoscopic thyroidectomy between January 2022 and December 2022 were enrolled in the study and randomly matched to patients with thyroid disease who underwent open thyroidectomy at the same time based on nodule size and pathology. Postoperative bleeding, hoarseness situation, hospital stay, postoperative drainage volume, laryngeal nerve palsy, hypoparathyroidism and wound infection were assessed in both groups.

**Results:**

Forty-four patients underwent endoscopic thyroidectomy successfully and two patients changed to open thyroidectomy. The amount of postoperative drainage for the endoscopic thyroidectomy group was 102.78 ± 28.04 mL, and which was 71.91 ± 19.20 for open thyroidectomy group (*p* < 0.001). The postoperative hospital stay for the endoscopic thyroidectomy group was 8.78 ± 2.57 days, and which was 7.22 ± 1.13 for open thyroidectomy group (*p* < 0.001). There was no significant difference in postoperative bleeding, hoarseness situation, and wound infection between the two groups. Laryngeal nerve palsy, supraclavicular nerve injury and hypoparathyroidism were not observed in any patient during this study.

**Conclusion:**

Chest-collarbone endoscopic thyroid surgery is acceptable. This treatment improves in a good cosmetic outcome in patients with thyroid disease. To assess patients with preoperative nodule size and nature of the case is the impact of the success rate, which is particularly important.

## Highlights

• Endoscopic thyroidectomy through chest-collarbone approach is safe and feasible.
• Chest-collarbone endoscopic thyroid surgery improves in a good cosmetic outcome.
• Preoperative evaluation of nodule size and nature affects the success rate of surgery.

## Introduction

Thyroid nodules are a common medical condition in Chinese people.^1^ At present, conventional open thyroidectomy is a common surgery method, and which is considered safe.^2^ However, thyroid nodules occur mostly in female patients and open thyroidectomy leaves an undesirable scar on neck. Endoscopic thyroidectomy has gained popularity for cosmetic reasons, compare with open thyroidectomy.^3^ Minimally invasive endoscopic thyroidectomy has become increasingly popular in thyroidectomy in Chinese.

Recently, endoscopic thyroidectomy has been performed in many centers via various routes, including the axillary, areola, and transoral approaches, with good evidence of its safety and feasibility for both benign and malignant thyroid tumors, but there are no guidelines regarding indications, or operative techniques.^4^, ^5^ It has been demonstrated that endoscopic thyroidectomy exhibits excellent therapeutic effects and improved cosmetic outcomes, compared with open thyroidectomy.^6^ Moreover, endoscopic thyroidectomy is also better for the improvement of postoperative quality of life than traditional open surgery, especially in scarring related problems, swallowing impairment and psychosocial impairment.^7^, ^8^, ^9^ However, the difficulty of operation and the wide range of surgical separation are still controversial areas in endoscopic thyroid surgery.

Several approaches have been developed to date, but chest-collarbone approach is easy to operation and have a good cosmetic outcome in Chinese.^10^ The chest-collarbone approach is a new approach for thyroidectomy and, the advantages and limitations of chest-collarbone approach and open thyroidectomy still have not been well understood. Therefore, we assessed the status of chest-collarbone endoscopic thyroidectomy procedures in Chinese, in terms of the advantages, disadvantages, complications, and limitations of this approach.

In this study, we conducted a comparative study on the feasibility, safety, and advantages of chest-collarbone thyroidectomy versus open thyroidectomy. A total of 92 patients with thyroid nodules admitted to our hospital between January 2022 and December 2022 were divided into chest-collarbone endoscopic thyroidectomy and control groups were randomly matched according to the pathology and nodule size. We then compared the safety and surgical outcomes between the two groups. Our findings may provide important guidelines for endoscopic thyroidectomy in the future.

## Methods

Subjects were recruited between January 2022 and December 2022. A total of 46 patients, who were diagnosed as thyroid nodules by clinical evaluation, underwent chest-collarbone endoscopic hemithyroidectomy with gasless were recruited to this study. Informed consent was obtained in all cases. A matched control patient was chosen during the same period. Chest-collarbone endoscopic thyroidectomy group patient was randomly matched with conventional open thyroidectomy patients by pathology and nodule size. No nerve integrity monitoring system was used to monitor the recurrent laryngeal nerve in any of the cases in this study. And all cases included were patients undergoing primary thyroidectomy. All patients’ demographic and clinical characteristics records were reviewed. All participants gave written informed consent, and the study was approved by the local ethics committee (nº 2023130201).

### Endoscopic procedure

**Figure 1:**
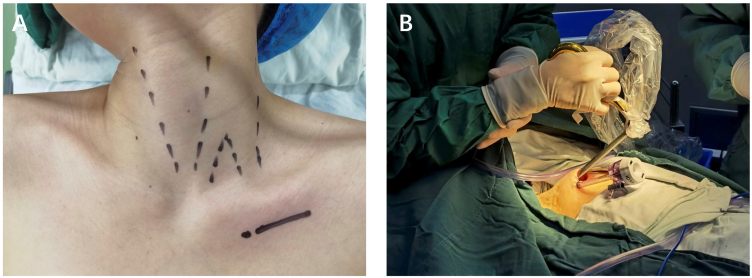
(A) Anatomical surface marking and incision design. (B) The trocars were punctured into the subcutaneous space.

**Figure 2:**
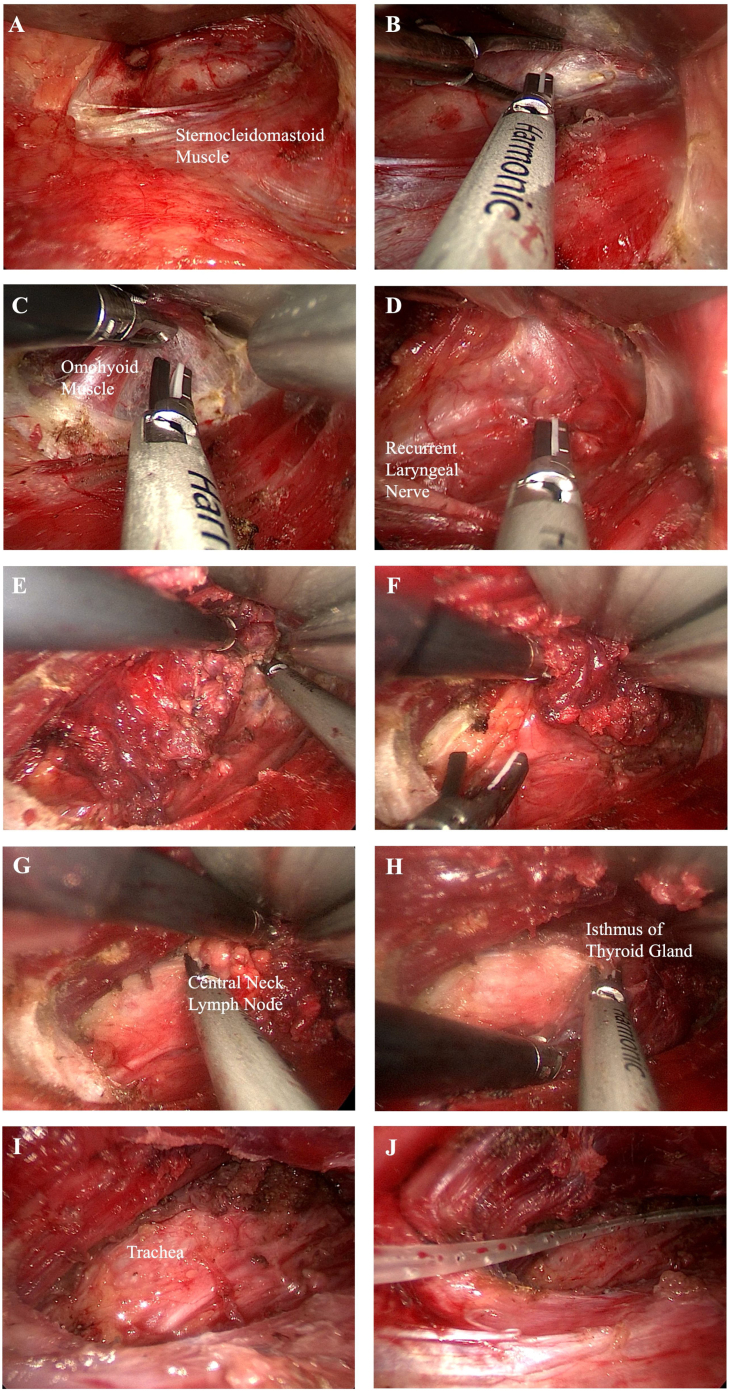
(A) A catheter reserve space was formed. (B) The sternocleidomastoid space was isolated and revealed. (C) The omohyoid muscle is exposed. (D) The recurrent laryngeal nerve was exposed. (E) An ultrasound knife was used to disentangle the superior thyroid. (F) Inferior thyroid tissue was freed. (G) The central lymph nodes were removed. (H) The isthmus of the thyroid gland was severed. (I) The thyroid lobe was completely removed. (J) Surgical wound drainage tube was placed.

Surgery was scheduled within one week after admission. All subjects underwent general anesthesia with endotracheal intubation and were placed in the supine position. The neck slightly turns to the opposite side of the surgery (Fig. 1). A monitor screen was placed at the head of the patient. As a control procedure, the chest-collarbone endoscopic thyroidectomy was performed as follows: a 5 cm incision was made along the subclavicle that penetrated the deep fascia. An electric knife was used to separate the subcutaneous space to form a catheter reserve space. After 5-mm incisions were made along the subclavian, a 5-mm trocar was placed into the subcutaneous loose connective tissue in the direction of the thyroid. Ultherapy was then employed to separate the subcutaneous loose connective tissue and moved sequentially along the lower side of the thyroid cartilage to the sternocleidomastoid. The anterior cervical muscles are separated from the side to expose the thyroid gland. An ultrasonic scalpel was used to free up the thyroid isthmus and ipsilateral thyroid lobe to sequentially expose the ipsilateral inferior thyroid artery and the middle thyroid vein so that the thyroid lesions can be removed. The specimen was pulled out through the laparoscopic port and a frozen section of the specimen was examined for pathologic confirmation. After no bleeding was found at the wound site, a suction drainage tube was placed to remove fluids that may build up in the areas of surgery (Fig. 2).

### Assessment of clinical outcomes

The following variables were examined: operation time, postoperative bleeding, hoarseness situation, postoperative hospital stay, postoperative drainage volume, wound infection, diameter of the resected thyroid nodule, permanent pathology, and total amount of drainage. The patients were examined for at least 1-month postoperatively for postoperative hematomas. The amount of drainage was measured over 24 h, and the drain was removed postoperatively if the quantity of drainage was less than 20 mL in 24 h.

Clinical symptoms and signs of hypocalcemia and serum calcium concentrations were assessed. Hypocalcemia was defined as a postoperative serum calcium level lower than 2.12 mmoL/L. Recurrent laryngeal nerve palsy and hypoparathyroidism were deemed permanent when no evidence of recovery was seen within 3-months postoperatively.

### Statistical analysis

All statistical analyses were conducted using the statistical program SPSS 20.0 for windows (SPSS, Chicago, IL, USA). The clinicopathologic variables were analyzed using two-tailed χ^2^ test and Student’s *t*-test.

## Results

There were 46 patients in the chest-collarbone endoscopic thyroidectomy group and 46 patients in the conventional open thyroidectomy group. Characteristics of those patients were shown in Table 1. Cases and controls were well matched. Postoperative pathology showed 18 nodular goiter, 2 follicular adenomas and 26 thyroid cancers in endoscopic thyroidectomy group. The mean nodule size was 1.7 ± 1.4 mm (Table 1).

**Table 1:** Baseline characteristics.

	Endoscopic thyroidectomy group (n = 46)	Open thyroidectomy group (n = 46)	*p*-value
**Age (years)**	32.7 ± 8.6	39.9 ± 8.8	<0.001
**Sex**			0.514
Female	31	28	
Male	15	18	
**Nodule size (cm)**	1.7 ± 1.4	2.0 ± 1.4	0.315
**Histology**			1.000
Nodular goiter	18	18	
Follicular adenomas	2	2	
Thyroid cancer	26	26	

Values were given as mean ± SD.

The amount of postoperative drainage for the endoscopic thyroidectomy group was 102.78 ± 28.04, which was 31 mL more than for the last 46 open thyroidectomy group (71.91 ± 19.20 mL) (Table 2). The postoperative hospital stay for the endoscopic thyroidectomy group was 8.78 ± 2.57 days, and which was 7.22 ± 1.13 for open thyroidectomy group (Table 3). There were two patients of conversion to open surgery during endoscopic thyroidectomy. Recurrent laryngeal nerve palsy and hypoparathyroidism were not observed in any patient during this study. No postoperative bleeding, hoarseness situation, or wound infection occurred in any patient during this study.

**Table 2:** The amount of postoperative drainage for the endoscopic thyroidectomy group and open thyroidectomy group.

	Endoscopic thyroidectomy group	Open thyroidectomy group	*p*
**Amount of postoperative drainage**	102.78 ± 28.04	71.91 ± 19.20	<0.001

**Table 3:** The hospital stay time for the endoscopic thyroidectomy group and open thyroidectomy group.

	Endoscopic thyroidectomy group	Open thyroidectomy group	*p*
**Postoperative hospital stay time (day)**	8.78 ± 2.57	7.22 ± 1.13	0.001

## Discussion

Several endoscopic thyroidectomy approaches have been developed to date, such as the transoral, areola, axilla-breast, and chest-collarbone approaches.^11^, ^12^ However, there is much debate and conflict about these procedures. For example, although the transoral thyroidectomy has the advantages of complete absence of scar on the body surface, one-time treatment of bilateral thyroid nodules, full exposure, and clearance of paratracheal low lymph nodes, etc., higher operational difficulty, longer learning curve, increased incidence of infection, and mental nerve injury are still the first problems of this surgery.^13^ At present, gasless transaxillary endoscopic thyroidectomy has been gradually accepted by surgeons because of its hidden incision and small influence on swallowing function.^14^ However, due to the long operation distance and the obstruction of the clavicular and sternoclavicular joints, endoscopic thyroidectomy through the axillary approach still has some defects, such as large subcutaneous separation range, high operation difficulty and difficulty in fully exposing the central region. As a result, many surgeons have shifted their attention to sternoclavicular approach thyroid surgery, which has the potential to ameliorate these defects due to its short anatomical distance and less resistance to clavicular and sternoclavicular joint obstruction. However, the safety and feasibility of the procedure remain controversial. In this study, we assessed the status of chest-collarbone thyroidectomy procedures in Chinese, in terms of the advantages, limitations, and complications of this approach.

In this study, recurrent laryngeal nerve palsy, supraclavicular nerve injury and hypoparathyroidism were not observed in any patient during this study. No postoperative bleeding, hoarseness situation, or wound infection occurred in any patient during this study. Thus breast-chest endoscopic thyroid surgery is acceptable. However, there were two patients of conversion to open surgery during endoscopic thyroidectomy. One case conversion to open surgery because of the nodule size was larger than 5 cm. For the indications and contraindications of endoscopic thyroidectomy, some paper responded that less than 1-cm single thyroid nodule was an adequate indication for endoscopic surgery,^15^ and others responded that less than 2-cm thyroid nodule was a possible indication,^16^, ^17^ but the last responded that if the tumor was located anteriorly, even more than 5 cm thyroid nodule could be a possible indication for an endoscopic thyroidectomy.^18^ In addition, we have also tried to remove bilateral thyroid nodules through unilateral incision, and the results are as expected, although the chest-collarbone approach can simultaneously treat part of the isthmic thyroid nodules through the traction of endoscopic instruments, it is still unable to treat the contralateral thyroid nodules, which is the same as the axillary approach. These results suggest that location and the size of thyroid nodules is a more important factor in determining whether endoscopic thyroidectomy is possible. Therefore, the accuracy of preoperative ultrasound assessment is particularly important for the screening of patients suitable for endoscopic thyroidectomy. For complications, such as laryngeal nerve palsy injury and hypoparathyroidism, most surgeons thought that there were not significant differences between conventional and endoscopic approaches.^19^, ^20^ However, in terms of serious complications of endoscopic thyroidectomy, many surgeons worried about injury to major vessels, such as the internal jugular vein and carotid artery during the dissection following thyroidectomy.^21^ In this study, our results suggested that laryngeal nerve palsy and hypoparathyroidism were not observed in any patient.

With the continuous development of minimally invasive surgical instruments, in recent years, Da Vinci robotic surgical system has begun to be used as a new technology in various approaches to thyroid surgery.^22^, ^23^ It is the most advanced control system for minimally invasive endoscopic surgical instruments, with unparalleled advantages such as filtering tremor and motion, high-resolution three-dimensional stereoscopic images, and remote control.^24^, ^25^ It provides a new choice for individual operation of patients. However, the lack of corresponding haptic feedback, the preoperative cavity construction and installation preparation time is too long, and the operation has a certain visual field blind area, which are also problems that beginners are easy to encounter. In addition, its high surgical cost, and the cost of regular maintenance of the surgical system largely limit its promotion. In short, there is no absolute advantage compared with the various endoscopic thyroid surgery or traditional open surgery that has been widely carried out. For a long time, it was just a new option for multiple thyroid surgery.

## Conclusion

In terms of the advantages, limitations, and complications of breast-chest endoscopic thyroidectomy. This treatment is acceptable and improves in a good cosmetic outcome in patients with thyroid disease. To assess patients with preoperative nodule size and nature of the case is the impact of the success rate, which is particularly important.

## Funding

This study was supported by grants from Zhejiang Science and Health Care Foundation of China (grant nº 2023KY1284), Jinhua nonprofit technology applied research projects of China (grant nº 2021-3-084), Jinhua public welfare technology application project research (grant nº 2023-4-076).

## Conflicts of interest

The authors declare no conflicts of interest.
