# Supplementary figures and images for: Endoscopic thyroidectomy via chest-collarbone approach versus conventional open thyroidectomy: a retrospective comparative study
Source: Braz J Otorhinolaryngol. 2024 Apr 3;90(4):101429. doi: 10.1016/j.bjorl.2024.101429 (PMC11050726; doi:10.1016/j.bjorl.2024.101429)

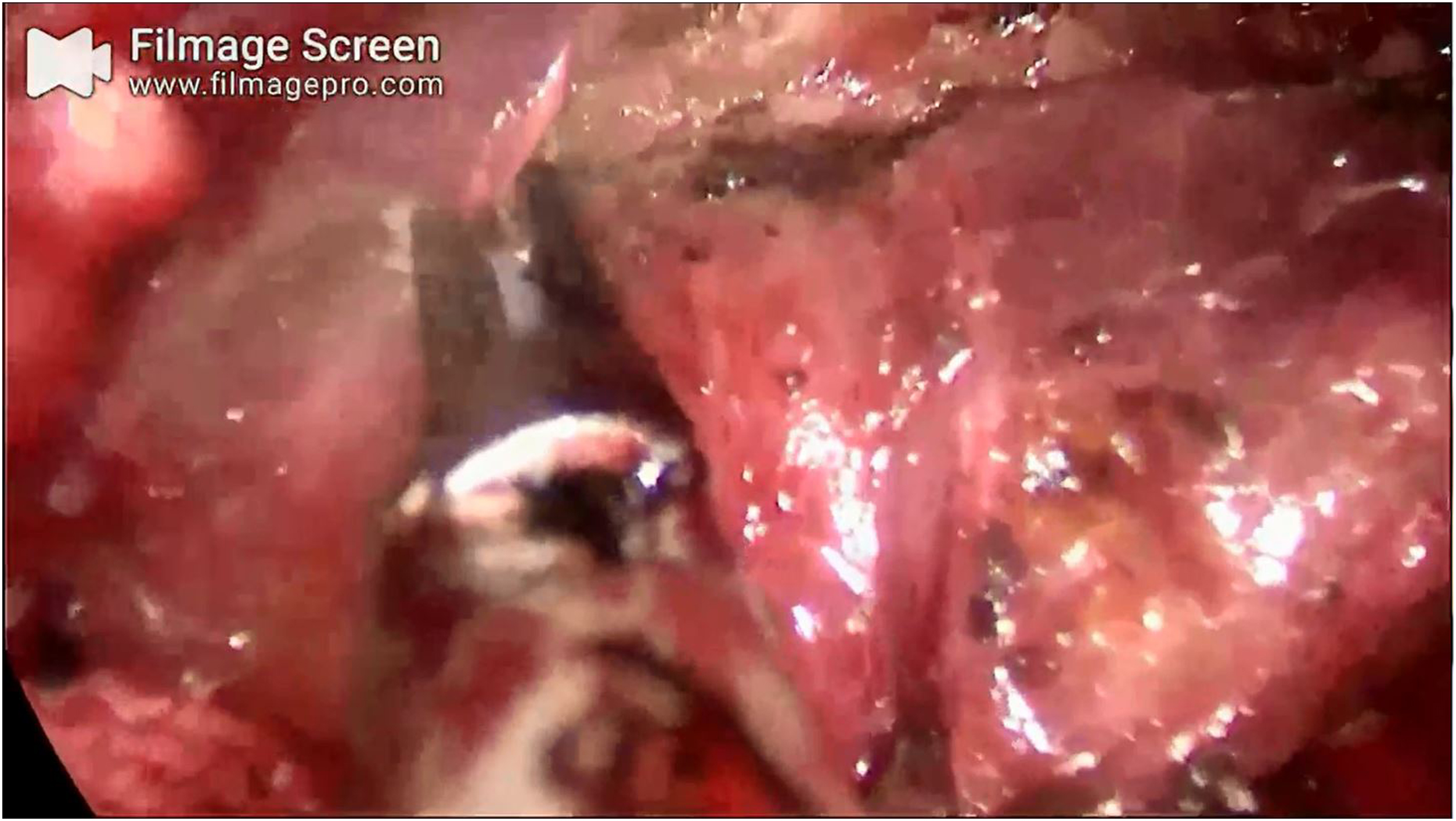

Supplement: Supplementary file 1 [file mmc1.zip › mmc1.jpg]
